# Exploring the cortical habituation in migraine patients based on contingent negative variation

**DOI:** 10.3389/fneur.2023.1226554

**Published:** 2023-08-30

**Authors:** Jinru Ning, Yongxiang Zhang, Yan Wang, Chang Liu, Yingying Cheng, Mingqin Zhu, Ming Dong, Xin Yang, Yudan Lv

**Affiliations:** ^1^Department of Neurology, The First Hospital of Jilin University, Changchun, China; ^2^Center on Aging Psychology, CAS Key Laboratory of Mental Health, Institute of Psychology, Beijing, China; ^3^Computer Science College, Dalian University of Technology, Dalian, China

**Keywords:** migraine, contingent negative variation (CNV), FFT (fast Fourier transform), cortical habituation, event-related potential (ERP)

## Abstract

**Introduction:**

Cognitive dysfunction has frequently been found in patients with migraine. The so-called contingent negative variation (CNV) and EEG power spectral densities may be the best choices to explore the underlining pathophysiology, such as cortical inhibition and habituation.

**Methods:**

Thirty migraine patients without aura and healthy controls matched for sex, age, and education were recruited separately for CNV recording. The amplitudes, latencies, and squares of different CNV components, such as oCNV, iCNV, tCNV, and PINV, were selected and analyzed. Behavioral data, such as manual reaction time (RT), were analyzed. We used the Person correlation coefficient R to analyze different ERP components in relation to clinical characteristics. A multiple regression analysis was conducted for the migraine group. Spectral analysis of EEG data from all channels using the fast Fourier transform (FFT).

**Results:**

The migraine group had longer A-latency, C-latency, and iCNV-latency than the control group. The migraine group had higher iCNV-amplitude, oCNV-amplitude, and tCNV-amplitude than the control group, especially those located in the occipital area. The iCNV-square, oCNV-square, tCNV-square, or PINV-square in the migraine group was significantly larger than the control group. Different correlations were found between clinical characteristics and ERP components. The delta or theta activity in the migraine group was statistically lower than in the control group.

**Discussion:**

Our study has revealed that migraine attacks may influence responsivity, pre-activation, habituation, and cortical inhibition not only on the behavioral level but also on the electrophysiological level. Abnormal changes in cortical habituation and inhibition can be interpreted as CNV components. Additionally, analyses have revealed correlations between CNV components and various factors, including age, the clinical course of the condition, attack frequency, pain intensity, and duration. Thus, repetitive migraine attacks can lead to a reduction in cortical inhibition and subsequent impairment in executive function.

## 1. Introduction

Migraine is a common chronic brain disorder characterized by recurrent throbbing headaches on one side or both sides. Migraine attacks are moderate to severe, lasting almost 4 to 72 h, and are often accompanied by autonomic neurological symptoms such as the fear of bright light or strong sounds, feelings of fatigue, nausea, and vomiting (1). According to different clinical features and the International Classification of Headache Disorders (ICHD-3β) guidelines (2), three sub-types of migraine can be classified: migraine with aura, migraine without aura, and chronic migraine.

In addition to headaches, migraine patients often complain of impaired concentration or reduced response after the attacks or in the interval of their headaches, which were called related cognitive dysfunctions. The Montreal Cognitive Scales (MoCA) and Mini-mental State Examination (MMSE) as assessment tools for rapid screening were commonly employed (3) but with higher false-negative rates and low sensitivity. Therefore, many researchers have recently been interested in using other tools to test the relationship between migraine and cognitive impairment, especially in migraine without aura as a common sub-type (4). Moreover, the event-related potential (ERP) as a cognitively evoked potential may be more objective in revealing how the cortex transmits and integrate (5). Among ERP paradigms, contingent negative variation (CNV) as a slow cortical event-related potential is gradually accepted to describe the correlates between neurobiology and cognitive activity (6).

In ERP studies, signal recording, noise removal, signal estimation, and signal analysis are of great importance. It should be kept in mind that recording clean EEG signals and dealing with various noises or unwanted artifacts are necessary. Then, based on these clean data, various techniques, such as spectral analysis and coherence analysis, are further performed. Therefore, proper data processing methods are crucial. Referring to Narayanan Srinivasan's methods of ERP analysis and spectral analysis (7, 8), we made some improvements in our methods.

Migraine has cortical inhibition dysfunction, which is thought to be associated with a reduced pre-activation level of the sensory cortex (9–11). The CNV data of migraine can be quantified and expressed as EEG power spectral density by fast Fourier transform (FFT) analysis (8). Thus, we applied FFT to present the topographic distribution of EEG power to compare the differences.

Moreover, other studies have found that cortical hyper-reactivity and a lack of habituation to repetitive stimuli seemed common in migraine (6). Habituation is the simplest form of implicit learning, which allows organisms to learn the characteristics of new stimuli. When a new stimulus appears, if it is irrelevant or not noxious, after successive exposures, an animal ignores it (12). Normal neuronal habituation is believed to play an important role in learning that allows the brain to deal with a new stimulation correctly in humans, so defective habituation and abnormal excitability in the cortex predicted cortical dysfunctions adapting to the environmental changes in migraine (12). Habituation can be studied in humans through evoked potential stimulation. Habituation of evoked potentials is manifested as a decrease in the amplitude of the evoked response after repeated stimulation (12). Thus, CNV combined with FFT may be helpful in meeting the goals of studies on cortical hyper-reactivity and cortical habituation in migraine patients.

The CNV was applied to study cortical habituation in migraine. The paradigm is a slow event-related potential developed between two related voice stimuli associated with complicated psychophysiological activities such as motivation, preparation, expectation, and attention (13, 14). The first voice is a warning stimulus (S1) to inform the subject of improving attention. The second voice serves as an imperative stimulus (S2), requiring the subject to provide a motor response (15). CNV consists of several components: (1) the initial CNV component (iCNV), measured as the amplitude maximum during the 500–1,000 ms after S1, has been associated with movement preparation (16) or an unspecific orienting response, (17) to improve attention to the following stimuli and prepare for a response as quick as possible before S2. (2) The terminal CNV component (tCNV), measured as mean amplitude within almost 200 ms before S2 onset, represents the stimulus anticipation (17). (3) The overall CNV (oCNV), measured as mean amplitude between S1 and S2, indicated the subjects' habituation to repetitive stimuli. In addition, the initial CNV component (iCNV) amplitudes and the terminal CNV component (tCNV) amplitudes depend on the excitability level of cortical pyramidal neurons (17). More researchers have found that migraine patients have, on average, higher negative amplitudes than healthy controls measured by CNV (18, 19). For example, in Kropp's study, migraine patients without aura had a significantly higher oCNV amplitude in the interval of migraine, suggesting that impaired habituation in migraine patients is evident during the pain-free intervals (19). Besides reflecting cortical excitability, CNV can also predict migraine attacks by observing CNV amplitude changes. Amplitude levels of CNV, especially the early component, show a negative upward trend 1 day before the attack, while the amplitude is normal 2–3 days after the attack, as Kropp's finding indicated (20).

Additionally, cortical excitability is an inappropriate activation of the central or peripheral nervous system, which can be quantified and presented as EEG power spectral density FFT.

Our study aims to detect cognitive dysfunction in migraine patients with the help of CNV and explore the underlying dysfunction of cortical inhibition and habituation. First, we compared the latency or amplitudes detected by CNV between migraine patients and healthy controls to evaluate whether migraine patients had delayed performance or over activity in the information process. Then, we interpreted such performance with the hypothesis of cortical reactivity, cortical inhibition, and habituation. Furthermore, we used CNV combined with FFT to observe regional or local abnormal changes, such as prefrontal, central, or others, to explore underlying cortical dysfunction caused by headache attacks. Thus, we may provide powerful evidence for cortical hyper-reactivity, decreased inhibition, and habituation in migraine patients with recurrent attacks, as testified by ERP with CNV.

## 2. Methods

The study was approved by the Ethics Committee of Jilin University First Hospital, and all participants provided signed informed consent.

### 2.1. Participants

Two groups of participants were recruited: (1) 30 migraine participants without aura from the headache clinic; and (2) 30 healthy volunteers matched in age, gender, and education. Diagnoses of migraine without aura were based on the ICHD-3βcriteria. Each patient had a headache diary and completed a structured questionnaire on demographics, including headache description, medical history, and medication. The headache description included the duration, severity, and frequency. Headache level was assessed using the Visual Analog Scale (VAS). Headache frequency was expressed as the number of headache attacks per month. All participants were asked to complete assessments, including the MMSE, MOCA, Hamilton Anxiety Scale (HAMA), and Hamilton Depression Scale (HAMD), and suspected participants were re-evaluated by psychiatrists in the following.

#### 2.1.1. Inclusion criteria

(1) Participants met the diagnostic criteria of migraine without aura according to the International Headache Disorders Third Edition (ICHD-3β); (2) migraine participants without aura were not accompanied by other headache disorders, such as cluster headaches, tension headaches, or other mental disorders; (3) all of them were between 30 and 70 years old, with a primary school education or above; (4) HAMA was not over 7; HAMD was not over 8; (5) no psychotropic drugs were used within 2 weeks; and (6) all of them were right-handed with no history of smoking, alcohol, or drug abuse.

#### 2.1.2. Exclusion criteria

(1) Headache was caused by organic diseases, severe anxiety, or depression; (2) participants had a neurological impairment, such as cerebral hemorrhage, brain trauma, a brain tumor, or previous brain surgery; (3) cognitive impairment was confirmed by MMSE or MOCA; (4) severe aural impairment; (5) participants who took psychotropic drugs or foods that might affect headache attacks during the study period, such as sedatives, anti-epileptic drugs, coffee, or tea; (6) participants who received prophylactic anti-migraine therapy; and (7) participants cannot fully cooperate to complete such a study.

### 2.2. Procedure

#### 2.2.1. ERP-recordings

All participants were tested by MMSE, MOCA, HAMA, and HAMD to exclude the possibility of cognitive impairment, anxiety, or depression before CNV, and migraines completed the Visual Analog Scale (VAS) (see Table 1). All migraineurs received CNV recordings during the inter-ictal phase. The CNV contained 40 trials involving a warning stimulus (S1), which lasted almost 1,000 ms in 50 HZ, and an imperative stimulus (S2) lasting up to 3,000 ms in 50 HZ. Subjects should enhance attention and make preparations when they hear the S1 and press the button at a fast speed after the S2 onset. The stimulus interval (ISI) has a duration of 3,000 ms calculated between S1 and S2. The duration of each trial is 5,000 ms, and the recordings started 200 ms before S1 and ended 4,500 ms after S2. During the CNV recording, all participants were seated in a comfortable armchair in a quiet, soundproof room with a suitable temperature. The process and the note were elaborated on in detail before CNV. The participants also had the opportunity to give it a try before beginning to complete such tasks with relaxation. The instructor asked participants to close their eyes before the onset of the S1.

**Table 1:** Clinical characteristics listed between migraine group and control group.

**Clinical characteristics**	**Migraine- group**	**Control- group**	***P***
Number of samples	30	30	-
Age (years)	36.83 ± 11.167	37.73 ± 11.685	0.761
Male (%)	43.3%	40%	-
Female (%)	56.7%	60%	-
Education	14.33 ± 3.898	13.43 ± 5.776	0.482
HAMA	3.70 ± 1.557	3.80 ± 1.400	0.795
HDMA	2.83 ± 1.642	3.40 ± 1.380	0.153
Pain intensity (VAS)	8.17 ± 1.487	-	-
Frequency of headache (per month)	14.23 ± 9.569	-	-
Drug using during study	-	-	-

HAMA, Hamilton Anxiety Rating Scale; HAMD, Hamilton Depression Rating Scale; VAS, Visual Analog Scale.

According to the International 10–20 System, the CNV data were recorded using Ag/AgC1 ring electrodes with bilateral mastoids as a reference. Besides, the impedances were kept below 10 k. An electrooculogram (EOG) recorded eye movements placed 1 cm below the left eye and 1 cm above the right eye. The recorded data were collected for 3 min.

#### 2.2.2. CNV-analysis

Pre-processing of CNV was conducted using Curry 7.0 software (Compumedics USA, Ltd., Charlotte, NC, USA). The data were bandpass filtered at 1–30 Hz. Any gross movement artifacts were removed from the recorded data by visual inspection, and eye blinks were removed using established mathematical procedures. Trials were rejected if they included significant physiological artifacts (amplitude exceeding ± 75 μV) at all cortical electrode sites. After artifact removal, a baseline correction was conducted by subtracting the mean value for 100 ms before the stimulus onset from the post-stimulus data for each trial.

**Figure 1:**
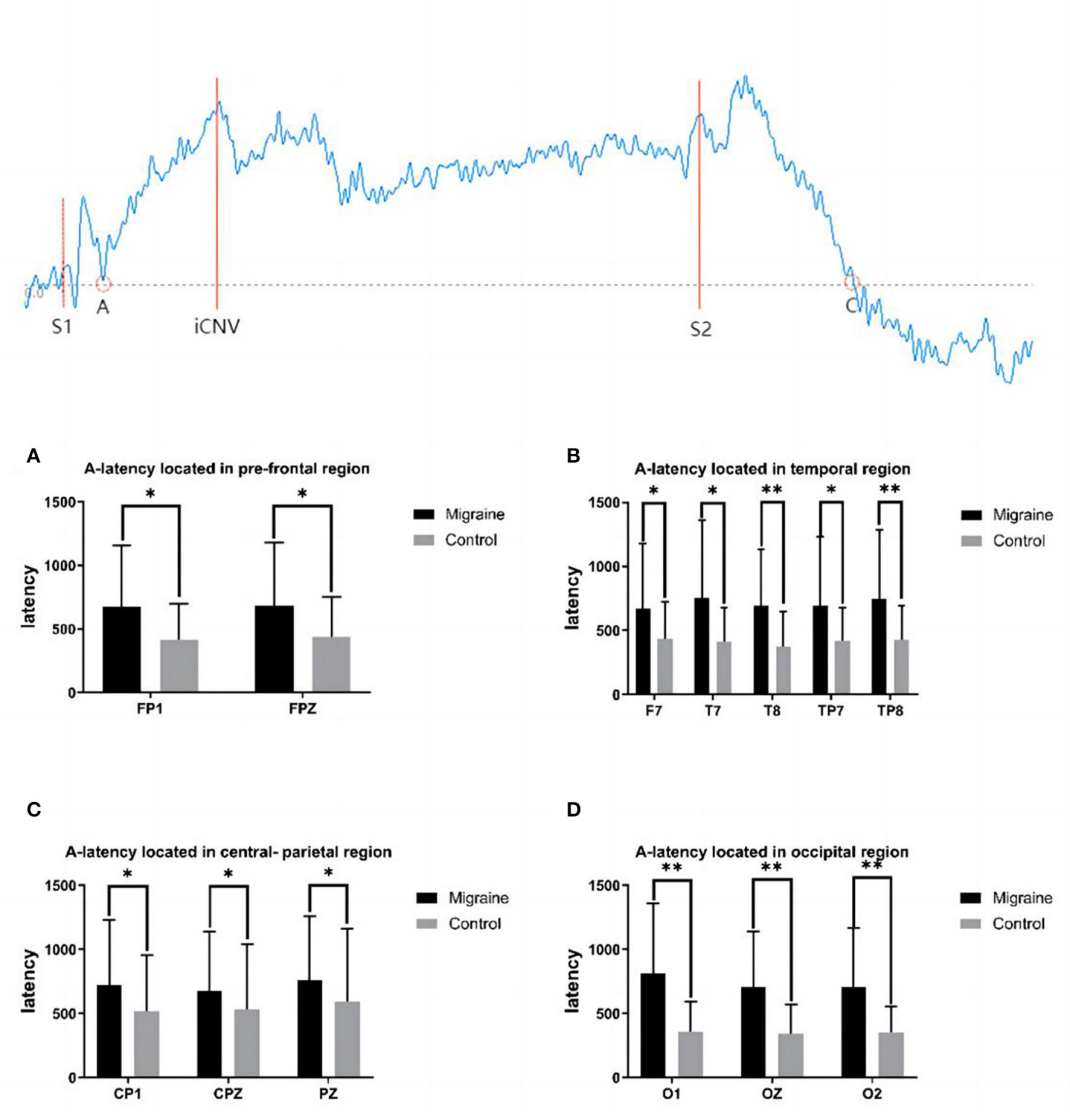
A-latency between the migraine and control groups located in different regions and a diagrammatic sketch of CNV. A significant difference in A-latency was found between the migraine and control groups (*P < 0.05, **P < 0.01).

We chose different CNV components to analyze, such as latencies, amplitudes, and squares of iCNV, tCNV, and oCNV. For amplitudes, the maximum amplitude of iCNV might be observed in 500–1,000 ms after S1—the iCNV, which is an averaged amplitude in the window of 200 ms around this maximum. The tCNV was the mean amplitude calculated at 200 ms before the onset of S2, and the mean amplitude between S1 and S2 was defined as the oCNV. For latencies, three were selected, which included A-latency, C-latency, and iCNV-latency. Determining the A, C, and iCNV points was important for calculating their latencies. The A point at which the wave group intersects the baseline as it deflects from the positive phase to the negative phase after S1 and A-latency is the time distance from S1 to the A point, as shown in Figure 1. The C point at which the wave group intersects the baseline as it deflects from negative phase to positive phase after S2 and C-latency is the time distance from S2 to the C point. iCNV-latency was the time distance from S1 to the iCNV point, and this point was in accord with the maximum amplitude of iCNV. For squares, we calculated the iCNV-square, the oCNV-square, the tCNV-square, and the PINV-square. The iCNV-square is the area of negative change from S1 to the iCNV point. The oCNV-square is the area of negative change from S1 to S2. The tCNV-square was the area of negative change 200 ms before the onset of S2. It is remarkable that PINV shows a significantly negative change from S2 to the C point. CNV-square is the average amplitude multiplied by the moment points in the time window and can be viewed as another measure of CNV amplitude to exclude potential interference. All components, including latency, amplitude, and square, were automatically analyzed and calculated using Curry 7.0 software. On behavioral indicators, reaction time (RT) was defined as the period between the onset of S2 and the pressing of the button.

#### 2.2.3. FFT analysis

Fast Fourier Transformation (FFT) was applied to complete spectral analysis on all channels (FP1, FP2, F3, F4, C3, C4, P3, P4, O1, O2, F7, F8, T3, T4, T5, and T6). The analysis was sampled at 200 Hz and filtered over the range of 0.16–70 Hz with a bandpass filter. After removing the artifacts of eye-related and motor-related movement, we set three spectrum powers: delta (1.5–4 Hz); theta (4–7.5 Hz); alpha (7.5–12.5 Hz); and beta (14–30 Hz) to analyze the EEG data. Statistical analysis was conducted using Matlab.

### 2.3. Statistical analysis

Statistical analysis was conducted using SPSS Statistics 26 with the help of professional statisticians. We tested the normal distribution of the CNV data and FFT data and conducted an independent sample *t*-test for homogeneity of variance. The statistically significant level was set at *p* < 0.05. In addition, a two-tailed Pearson's R correlation was conducted between CNV parameters and migraine characteristics such as education, course time, duration, frequency, and pain severity.

## 3. Results

### 3.1. Clinical evaluation

There was no difference in age, education, gender, or the score of HAMA, or HAMD, between the migraine group and the control group, as listed in Table 1.

### 3.2. Behavioral performance

No statistical differences have been found in both groups with a high hit rate in response to S2 (controls: 100% vs. migraine: 100%).

There was no difference between the migraine and the control groups in reaction times (RT) to S2 (Migraine: 287.81 ± 45.59 ms vs. Controls: 292.57 ± 41.05 ms, *P* > 0.05).

### 3.3. CNV-latency between the migraine group and the control group

#### 3.3.1. A-latency

A-point has been selected as the transforming point from the positive phase to the negative phase after S1 between the baseline and the CNV wave. A-latency was defined as the time from S1 to the A point. A statistical difference in A-latency between groups located in various areas was found. In the pre-frontal region, the A-latency of the migraine group located in FP1 and FPZ was longer than the controls (FP1: 672.700 ± 482.383 ms vs. 418.000 ± 278.286 ms, *P* = 0.017; FPZ: 684.600 ± 494.060 ms vs. 439.930 ± 314.251 ms, *P* = 0.020). In the temporal region, the A-latency of the migraine group located in F7, T7, T8, TP7, and TP8 was longer than the control group, respectively (F7: 671.300 ± 505.033 ms vs. 438.730 ± 283.343 ms, *P* = 0.030; T7: 753.600 ± 610.481 ms vs. 413.170 ± 263.588 ms, *P* = 0.018; T8: 697.130 ± 437.993 ms vs. 374.930 ± 272.899 ms, *P* = 0.001; TP7: 694.400 ± 535.641 ms vs. 422.630 ± 254.787 ms, *P* = 0.025; TP8: 746.700 ± 536.882 ms vs. 426.570 ± 268.756 ms, *P* = 0.005). In the central-parietal region, the A-latency of the migraine group located in CP1, CPZ, and PZ was longer than the control group, respectively (CP1: 725.970 ± 502.350 ms vs. 517.270 ± 436.607 ms, *P* = 0.024; CPZ:678.370 ± 461.907 ms vs. 532.000 ± 511.222 ms, *P* = 0.048; PZ: 762.667 ± 495.406 ms vs. 592.540 ± 568.487 ms, *P* = 0.046). In the occipital region, the A-latency of the migraine group located in O1, OZ, and O2 was longer than the control group, respectively (O1:813.780 ± 545.497 ms vs. 360.533 ± 231.115 ms, *P* < 0.001; OZ:705.870 ± 437.765 ms vs. 344.130 ± 226.813 ms, *P* < 0.001; O2: 704.610 ± 462.552 ms vs. 353.967 ± 199.009 ms, *P* < 0.001).

As shown above, the migraine group has a longer A-latency located in the pre-frontal, central-parietal, temporal, or occipital area than the control group, as shown in Figure 1.

#### 3.3.2. C-latency

The C point was selected as the transforming point from the negative phase to the positive phase after S2. The C-latency is the time from S2 to the C point. There was a significant statistical difference between the migraine group and the control group distributed across all channels, with much longer latency in the migraine group. In the pre-frontal region, there was a significant difference in C-latency in FP1, FPZ, and FP2 between the migraine group and the control group (FP1: 2,023.380 ± 1,002.054 ms vs. 761.467 ± 473.383 ms, *P* < 0.001; FPZ: 2,096.320 ± 998.117 ms vs. 776.833 ± 509.381 ms, *P* < 0.001; FP2: 2,249.260 ± 914.454 ms vs. 774.433 ± 548.543 ms, *P* < 0.001). In the frontal region, there was a significant difference in C-latency in F3, FZ, and F4 between the migraine group and the control group (F3: 2,185.987 ± 975.575 ms vs. 961.173 ± 585.460 ms, *P* < 0.001; FZ: 2,106.860 ± 1,016.965 ms vs. 872.713 ± 535.066 ms, *P* < 0.001; F4: 2,022.440 ± 699.720 ms vs. 927.980 ± 551.856 ms, *P* < 0.001). In the frontal-central region, there was a significant difference in C-latency in FC1, FCZ, and FC2 between the migraine group and the control group (FC1: 1,848.280 ± 783.395 ms vs. 942.533 ± 523.046 ms, *P* < 0.001; FCZ: 1,973.200 ± 791.625 ms vs. 808.870 ± 509.074 ms, *P* < 0.001; FC2: 1,940.807 ± 692.340 ms vs. 871.627 ± 585.499 ms, *P* < 0.001). In the central region, there was a significant difference of C-latency in C3, CZ, C4, CP1, CPZ, and CP2 between the migraine group and the control group (C3: 1,909.510 ± 747.032 ms vs. 981.127 ± 524.333, *P* < 0.001; CZ: 1,925.633 ± 761.057 ms vs. 865.817 ± 516.142 ms, *P* < 0.001; C4: 2,044.033 ± 754.232 ms vs. 945.363 ± 548.634 ms, *P* < 0.001; CP1: 1,863.933 ± 764.085 ms vs. 762.860 ± 497.511 ms, *P* < 0.001; CPZ: 1,904.030 ± 791.050 ms vs. 790.030 ± 499.867 ms, *P* < 0.001; CP2: 1,919.067 ± 731.378 ms vs. 886.073 ± 579.597 ms, *P* < 0.001). In the parietal region, there was a significant difference in C-latency in P3, PZ, and P4 between the migraine group and the control group (P3: 2,117.867 ± 653.170 ms vs. 1,174.473 ± 684.697 ms, *P* < 0.001; PZ: 2,126.067 ± 625.084 ms vs. 994.933 ± 674.685 ms, *P* < 0.001; P4: 2,043.193 ± 658.508 ms vs. 1,048.600 ± 687.651 ms, *P* < 0.001). In the temporal region, there was a significant difference in C-latency in F7, F8, T7, T8, TP7, and TP8 between the migraine group and the control group (F7: 2,165.200 ± 938.777 ms vs. 825.867 ± 558.901 ms, *P* < 0.001; F8: 2,061.567 ± 732.593 ms vs. 805.367 ± 525.782 ms, *P* < 0.001; T7: 2,073.887 ± 683.060 ms vs. 801.400 ± 460.858 ms, *P* < 0.001; T8: 1,892.940 ± 719.592 ms vs. 767.933 ± 493.722 ms, *P* < 0.001; TP7: 1,949.200 ± 781.246 ms vs. 61.040 ± 416.691 ms, *P* < 0.001; TP8: 1,982.613 ± 772.013 ms vs. 826.100 ± 629.138 ms, *P* < 0.001). In the occipital region, there was a significant difference in O1, OZ, and O2 between the migraine group and the control group (O1: 2,023.113 ± 608.460 ms vs. 793.333 ± 619.650 ms, *P* < 0.001; OZ: 2,065.607 ± 697.384 ms vs. 770.600 ± 614.614 ms, *P* < 0.001; O2: 2,038.280 ± 679.325 ms vs. 766.000 ± 611.930 ms, *P* < 0.001).

**Figure 2:**
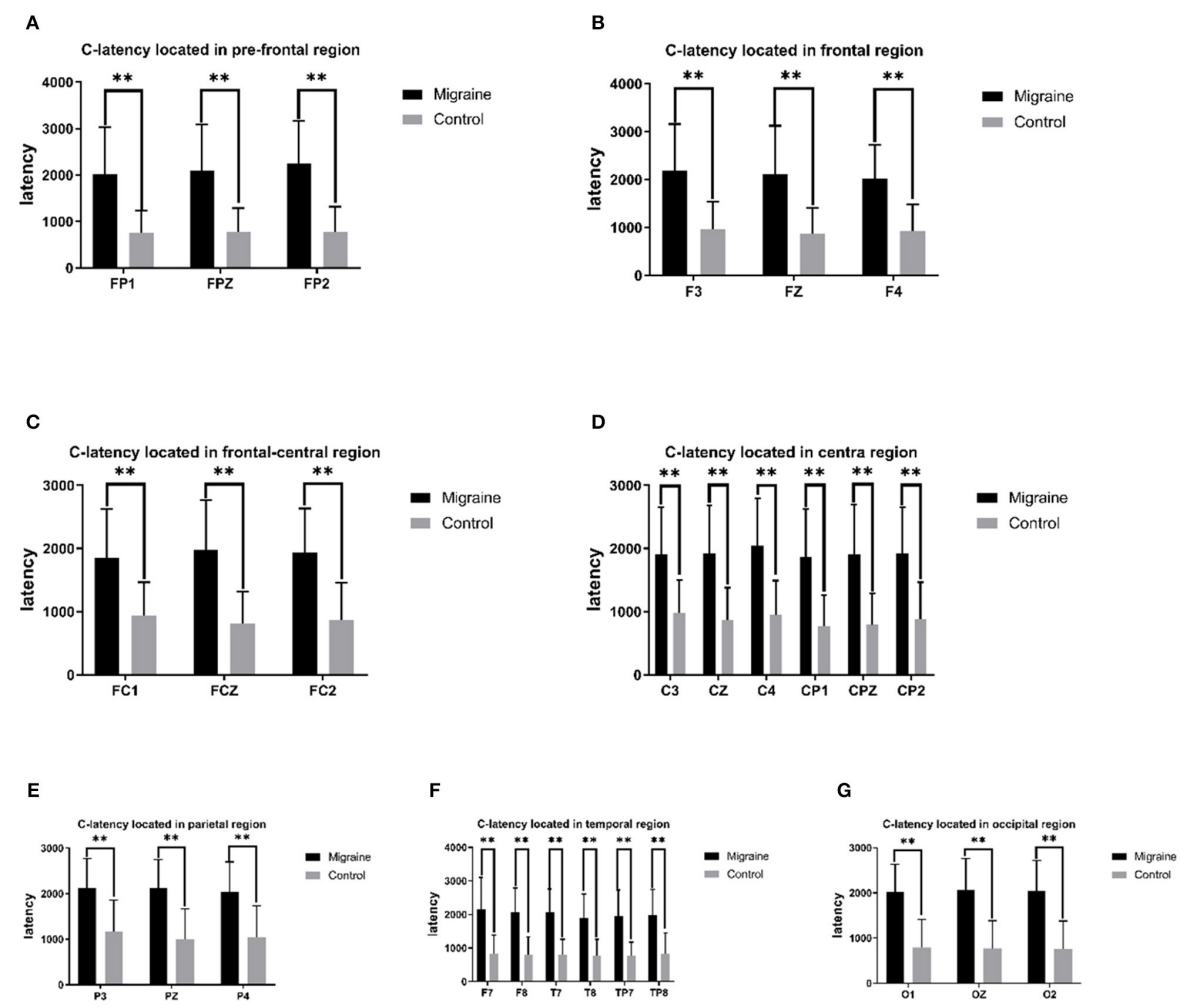
C-latency between the migraine group and the control group is located in different regions. A significant difference in C-latency was found between the migraine and control groups (*P < 0.05, **P < 0.01).

As shown above, the migraine group has a longer C-latency located in the pre-frontal, central-parietal, temporal, or occipital area than the control group, as shown in Figure 2.

#### 3.3.3. iCNV-latency

The initial CNV (iCNV) was defined as the maximum amplitude during the 500–1,000 ms after S1, and the iCNV-latency was defined as the time from the S1 onset to the maximum amplitude of iCNV. The iCNV-latency of the migraine group located in prefrontal, frontal, central, and temporal areas was statistically longer than controls. In the prefrontal region, the iCNV-latency of the migraine group was longer in FP1, FPZ, and FP2, respectively (FP1: 670.630 ± 78.003 ms vs. 621.330 ± 76.504 ms, *P* = 0.007; FPZ: 681.970 ± 70.78 ms vs. 626.300 ± 76.574 ms, *P* = 0.002; FP2: 682.030 ± 74.088 ms vs. 616.100 ± 75.084 ms, *P* = 0.001). In the frontal region, the iCNV-latency of the migraine group in F3, FZ, and F4 was longer, respectively (F3: 667.670 ± 75.617 ms vs. 630.730 ± 73.832 ms, *P* = 0.027; FZ: 664.730 ± 75.532 ms vs. 625.470 ± 71.447 ms, *P* = 0.027; F4: 666.430 ± 70.952 ms vs. 630.230 ± 76.517 ms, *P* = 0.033). In the central region, the iCNV-latency of the migraine group in FC2, C3, and C4 was longer, respectively (FC2: 666.900 ± 66.191 ms vs. 631.930 ± 73.555 ms, *P* = 0.025; C3: 682.170 ± 66.927 ms vs. 650.430 ± 69.138 ms, *P* = 0.047; C4: 680.670 ± 62.613 ms vs. 645.000 ± 72.756 ms, *P* = 0.031). In the temporal region, the iCNV-latency of the migraine group in F7, F8, T7, T8, and TP8 was longer, respectively (F7: 676.830 ± 68.017 ms vs. 634.470 ± 74.112 ms, *P* = 0.019; F8: 674.230 ± 72.304 ms vs. 628.200 ± 72.169 ms, *P* = 0.009; T7: 676.770 ± 69.895 ms vs. 642.000 ± 68.259 ms, *P* = 0.043; T8: 676.770 ± 64.591 ms vs. 639.500 ± 68.017 ms, *P* = 0.017; TP8: 677.970 ± 59.719 ms vs. 639.430 ± 71.467 ms, *P* = 0.038).

**Figure 3:**
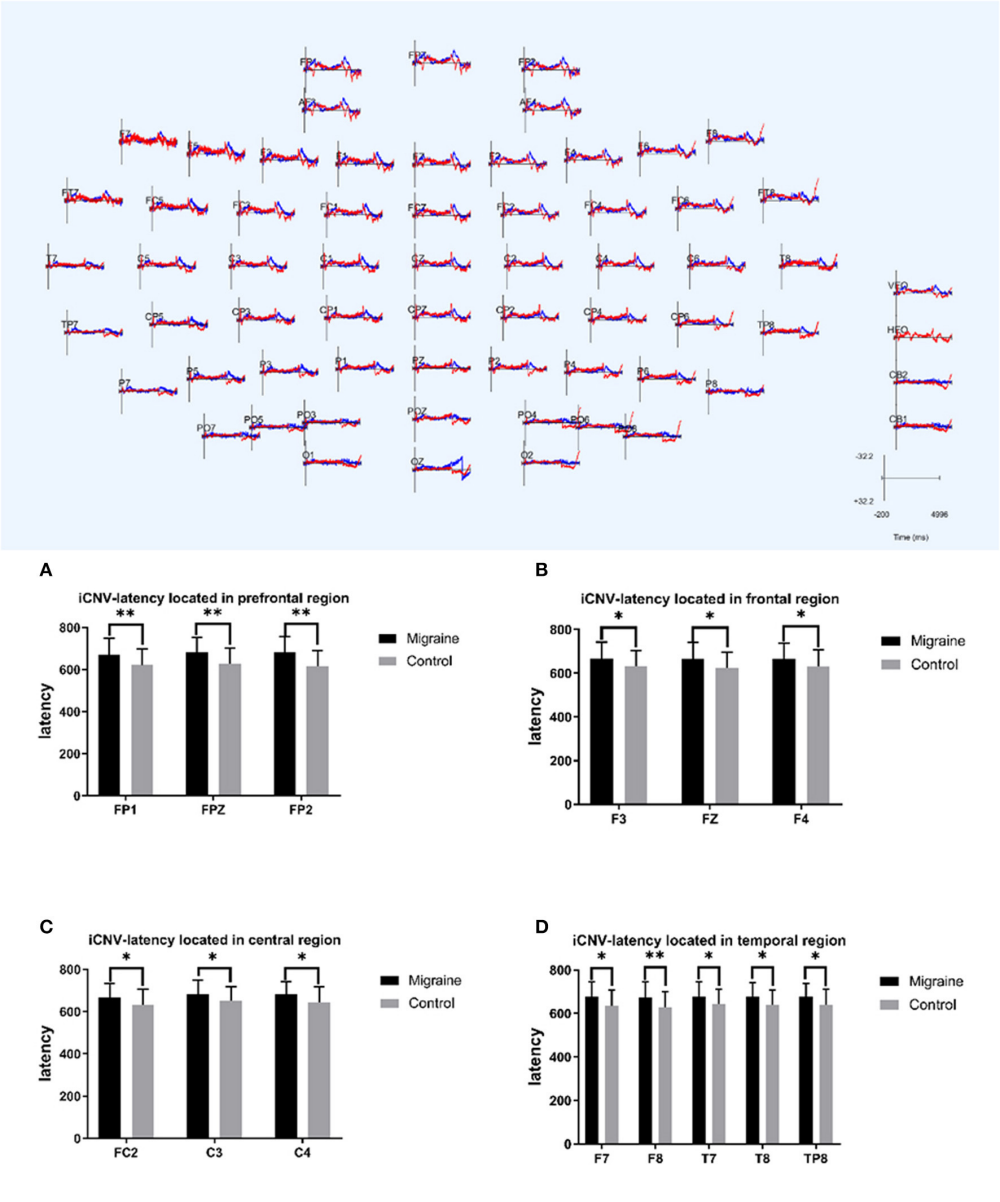
iCNV-latency between migraine and control groups located in different regions. A significant difference in iCNV-latency was found between the migraine and control groups (*P < 0.05, **P < 0.01).

As shown above, the migraine group has longer iCNV-latency located in the pre-frontal, frontal, central, and temporal areas than the control group, as shown in Figure 3.

### 3.4. CNV-amplitude between the migraine group and the control group

#### 3.4.1. iCNV-amplitude

The initial CNV (iCNV) occurs at 200 ms around the individual maximum amplitude during 500–1,000 ms after S1. The iCNV-amplitude of the migraine group located in the occipital region (O1, O2) was higher (much more negative) than the control group, respectively (O1: 59.408 ± 119.895 Uv vs. 38.324 ± 105.087 Uv, *P* = 0.041; O2: 13.766 ± 20.977 Uv vs. 9.566 ± 30.769 Uv, *P* = 0.011).

#### 3.4.2. oCNV-amplitude

The overall CNV (oCNV) is the average amplitude between S1 and S2. The oCNV-amplitude of the migraine group located in P4, O1, and O2 was higher (much more negative) than the control group, respectively (P4: 16.365 ± 25.202 Uv vs. 14.350 ± 37.132 Uv, *P* = 0.032; O1: 51.205 ± 123.043 Uv vs. 23.594 ± 55.115 Uv, *P* = 0.032; O2: 16.793 ± 27.275 Uv vs. 13.350 ± 37.625 Uv, *P* = 0.033).

#### 3.4.3. tCNV-amplitude

The terminal CNV (tCNV) is the average amplitude detected 200 ms before S2 onset. Differences in tCNV-amplitude have been found in P4 or O1 between the migraine group and the control group (P4:26.642 ± 39.382 Uv vs. 13.421 ± 15.239 Uv, *P* = 0.029; O1: 84.706 ± 202.765 Uv vs. 47.967 ± 120.831 Uv, *P* = 0.014).

**Figure 4:**
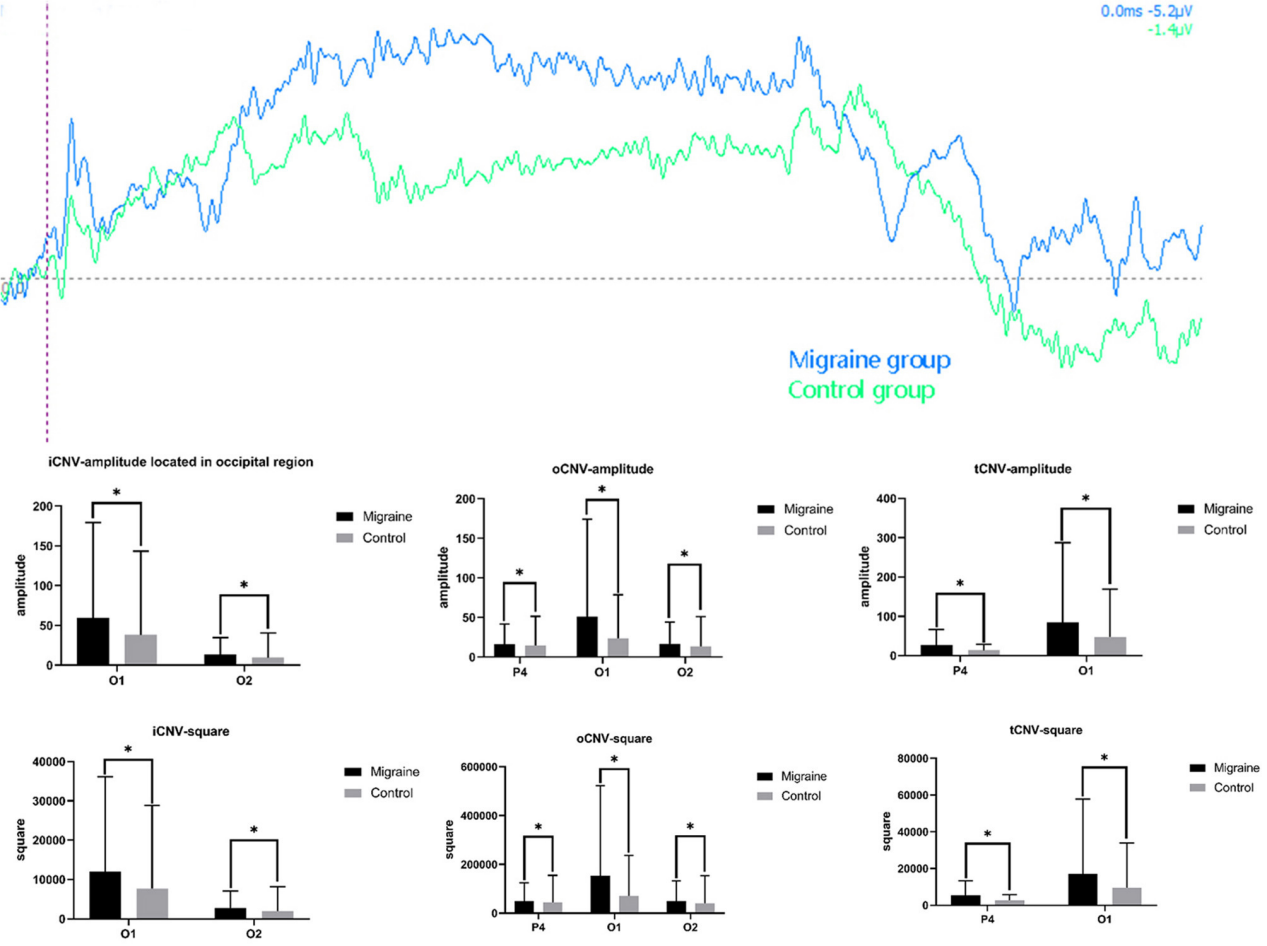
Different amplitude and square components between the migraine and control groups. A significant difference in CNV amplitude was found between the migraine and control groups (*P < 0.05, **P < 0.01). A significant difference in CNV-square was found between the migraine and control groups (*P < 0.05, **P < 0.01).

As shown above, the migraine group has higher iCNV-amplitude, oCNV-amplitude, and tCNV-amplitude than the control group, especially in the occipital area, as shown in Figure 4.

### 3.5. CNV-square

#### 3.5.1. iCNV-square

The iCNV-square is the area of negative change from S1 to the iCNV point. There was a statistical difference in iCNV-square between the migraine and control groups. The iCNV-square of the migraine group located in the occipital area (O1 or O2) was significantly larger than the control group, respectively (O1: 11,954.925 ± 24,121.964 Uvms vs. 7,708.913 ± 21,122.061 Uvms, *P* = 0.043; O2: 2,770.694 ± 4,233.389 Uvms vs. 1,925.650 ± 6,185.032 Uvms, *P* = 0.011).

#### 3.5.2. oCNV-square

The oCNV-square is the area of negative change from S1 to S2. There was a statistical difference in the oCNV-square between the migraine and control groups. The oCNV-square of the migraine group located in the parietal-occipital area (P4, O1, or O2) was significantly larger than the control group (P4: 49,114.777 ± 75,653.913 Uvms vs. 43,069.951 ± 111436.295 Uvms, *P* = 0.032; O1: 153,672.165 ± 369,256.752 Uvms vs. 70,810.608 ± 165,398.981 Uvms, *P* = 0.032; O2: 50,400.226 ± 81,875.971 Uvms vs. 40,068.149 ± 112,915.586 Uvms, *P* = 0.033).

#### 3.5.3. tCNV-square

The tCNV-square is the area of negative change 200 ms before the onset of S2. There was a statistical difference in tCNV-square between the migraine and control groups. The tCNV-square of the migraine group located in the occipital area (P4 or O1) was significantly larger than the control group (P4: 26.642 ± 39.382 Uvms vs. 13.421 ± 15.239 Uvms, *P* = 0.029; O1: 84.706 ± 202.765 Uvms vs. 47.967 ± 120.831 Uvms, *P* = 0.014), shown as Figure 4.

#### 3.5.4. PINV-square

**Figure 5:**
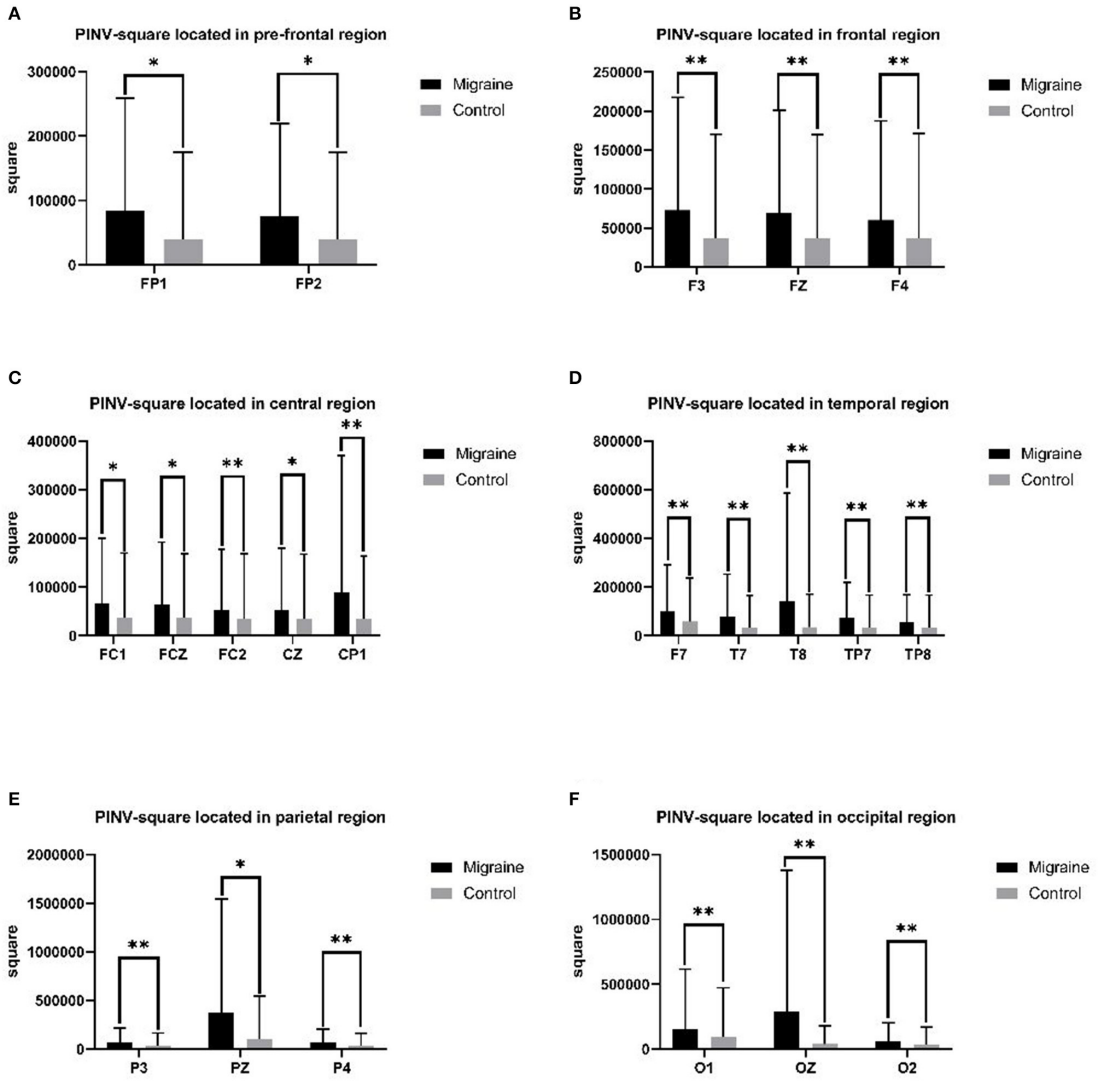
PINV-square between the migraine and control groups located in different regions. A significant difference in PINV-square was found between the migraine and control groups (*P < 0.05, **P < 0.01).

PINV-square was defined as the area in the post-imperative negative variation (PINV) compared to the baseline. A different PINV-square was statistically analyzed between the migraine group and the control group. In the prefrontal-frontal region, the PINV-square of the migraine group located in FP1, FP2, F3, FZ, and F4 was significantly larger than the control group (FP1: 83,712.475 ± 175,519.711 Uvms vs. 39,976.680 ± 135,038.827 Uvms, *P* = 0.028; FP2: 75,996.106 ± 143,368.343 Uvms vs. 39,263.931 ± 135,157.485 Uvms, *P* = 0.026; F3: 72,724.364 ± 144,734.927 Uvms vs. 36,695.630 ± 133,626.692 Uvms, *P* = 0.008; FZ: 68,944.736 ± 131,823.649 Uvms vs. 36,840.289 ± 133,069.578 Uvms, *P* = 0.010; F4: 60,125.339 ± 127,267.327 Uvms vs. 37,167.643 ± 133,922.177 Uvms, *P* = 0.004). In the frontal-central region, the PINV-square of the migraine group located in FC1, FCZ, FC2, CZ, and CP1 was significantly larger than the control group (FC1: 66,323.723 ± 133,497.434 Uvms vs. 36,801.039 ± 133,075.002 Uvms, *P* = 0.041; FCZ: 64,267.322 ± 127,939.905 Uvms vs. 37,198.558 ± 132,121.786 Uvms, *P* = 0.044; FC2: 53,613.937 ± 124,024.052 Uvms vs. 35,565.573 ± 132,708.319 Uvms, *P* = 0.006; CZ: 51,958.663 ± 127,849.090 Uvms vs. 35,290.215 ± 132,108.437 Uvms, *P* = 0.046; CP1: 88,847.141 ± 281,280.697 Uvms vs. 34,231.995 ± 128,917.592 Uvms, *P* = 0.009). In the temporal region, the PINV-square of the migraine group located in F7, T7, T8, TP7, and TP8 was significantly larger than the control group (F7: 99,171.386 ± 191,170.107 Uvms vs. 58,457.103 ± 178,155.863 Uvms, *P* = 0.005; T7: 80,661.655 ± 172,382.346 Uvms vs. 33,039.721 ± 133,094.549 Uvms, *P* = 0.010; T8: 140,533.512 ± 444,262.338 Uvms vs. 35,711.136 ± 135,160.275 Uvms, *P* = 0.007; TP7: 74,712.097 ± 145,802.494 Uvms vs. 33,158.506 ± 133,789.831 Uvms, *P* = 0.002; TP8: 55,287.648 ± 112,453.021 Uvms vs. 33,288.560 ± 133,728.702 Uvms, *P* = 0.007). In the parietal region, there was a significant difference in PINV-square in P3, PZ, and P4 between the migraine group and the control group (P3: 71,674.899 ± 142,324.050 Uvms vs. 34,235.916 ± 131,668.484 Uvms, *P* = 0.007; PZ: 377,178.116 ± 167,264.628 Uvms vs. 99,827.967 ± 445,397.007 Uvms, *P* = 0.014; P4: 65,912.917 ± 137,864.210 Uvms vs. 32,961.775 ± 131,056.177 Uvms, *P* = 0.001). In the occipital region, the PINV-square of the migraine group located in O1, OZ, and O2 was significantly larger than the control group (O1: 157,433.124 ± 459,465.915 Uvms vs. 91,364.421 ± 381,590.946 Uvms, *P* < 0.001; OZ: 290,525.282 ± 1,085,241.641 Uvms vs. 38,842.452 ± 143,235.764 Uvms, *P* = 0.004; O2: 61,725.662 ± 140,221.871 Uvms vs. 33,086.561 ± 135,497.122 Uvms, *P* = 0.003), shown as Figure 5.

### 3.6. Evaluation between clinical characteristics and CNV

#### 3.6.1. Evaluation between A-latency and clinical course duration

**Figure 6:**
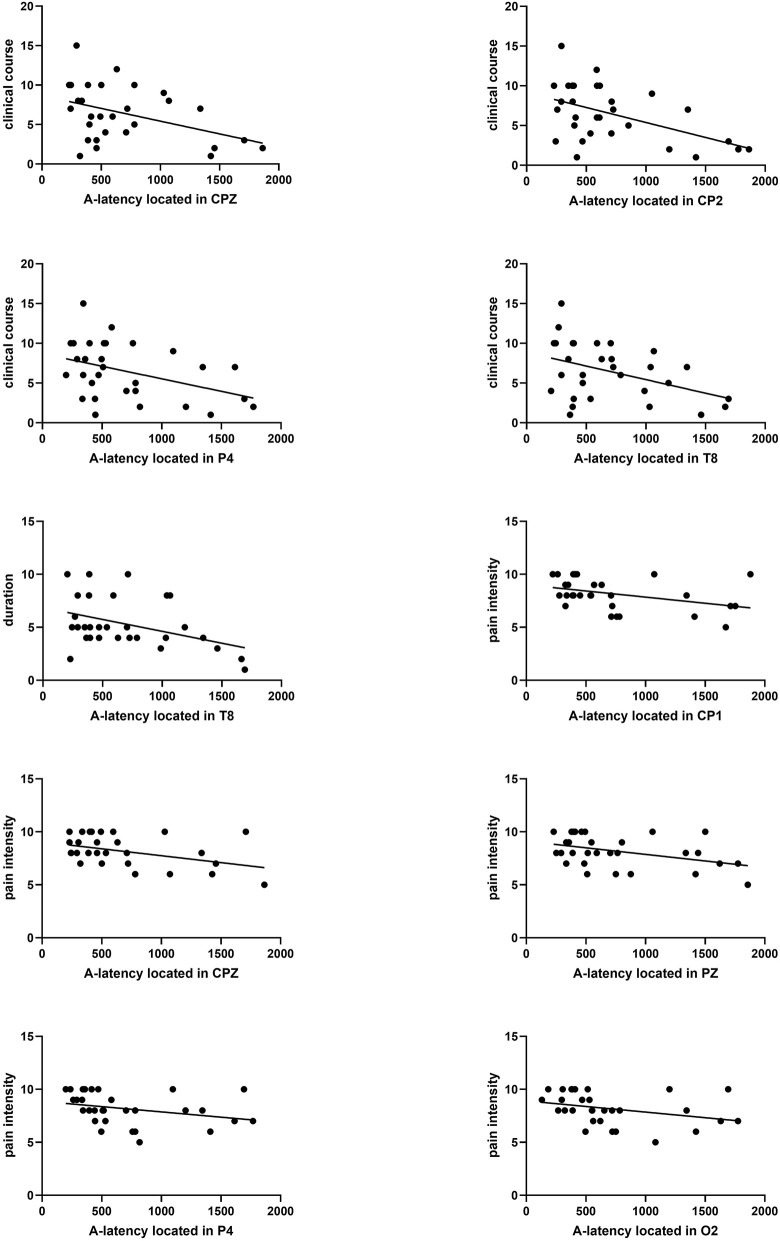
Correlation analysis between A-latency and clinical course, duration, and pain severity. A significant correlation between clinical course and A-latency located in CPZ, CP2, P4, or T8 was found (*P* = 0.031 r = −0.393, *P* = 0.010 r = −0.464, *P* = 0.042 r = −0.373, *P* = 0.040 r = −0.377). Significant correlation between duration and A-latency located in T8 was found (*P* = 0.036 r = −0.384). Significant correlation between pain severity and A-latency located in CP1, CPZ, PZ, P4, or O2 was found (*P* = 0.014 r = −0.443, *P* = 0.038, r = −0.381, *P* = 0.038 r = −0.381, *P* = 0.006 r = −0.489, *P* = 0.008 r = −0.476).

The clinical course was calculated by “years” from the first migraine attack to the present. In our study, the clinical course of migraine was significantly correlated with A-latency in the parietal area (CPZ, CP2, and P4) or temporal area (T8). The Pearson coefficient was presented as *P* = 0.031 r = −0.393; *P* = 0.010 r = −0.464; *P* = 0.042 r = −0.373; *P* = 0.040 r = −0.377. In addition, the duration means the average duration of migraine attacks in the past year, which was correlated with A-latency in T8, presented as *P* = 0.036 r = −0.384 (Figure 6).

#### 3.6.2. Evaluation between A-latency and pain intensity

There was a significant negative correlation between pain intensity and A-latency of CNV distributed throughout the parietal region (CP1, CPZ, PZ, and P4), which was presented as *P* = 0.014 r = −0.443; *P* = 0.038, r = −0.381; *P* = 0.038 r = −0.381; and *P* = 0.006 r = −0.489, respectively (Figure 6).

#### 3.6.3. Evaluation between C-latency and migraine duration or education

**Figure 7:**
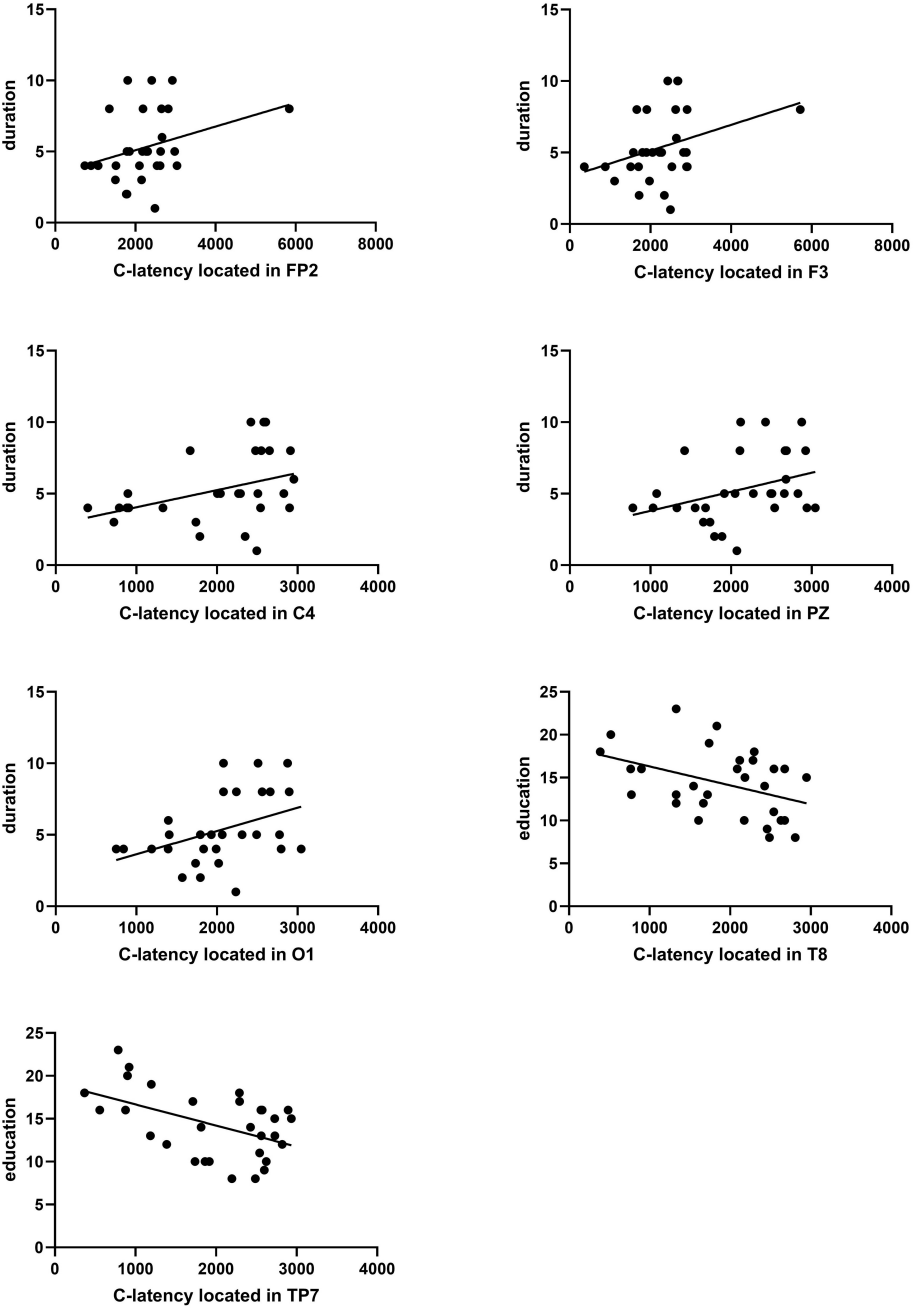
Correlation analysis between C-latency and duration in education. A significant correlation between duration and C-latency located in FP2, F3, C4, PZ, or O1 was found (*P* = 0.029 r = 0.399, *P* = 0.040 r = 0.376, *P* = 0.010 r = 0.465, *P* = 0.025 r = 0.408, *P* = 0.016 r = 0.437). A significant correlation between education and C-latency located in T8 or TP7 was found (*P* = 0.035 r = −0.386, *P* = 0.023 r = −0.415).

The duration of migraine was positively correlated with C-latency, and educational level was negatively correlated with C-latency. The Pearson coefficient between duration and C-latency located in FP2, F3, C4, PZ, and O1 was presented as *P* = 0.029 r = 0.399; *P* = 0.040 r = 0.376; *P* = 0.010 r = 0.465; *P* = 0.025 r = 0.408; *P* = 0.016 r = 0.437 (Figure 7). The Pearson coefficient between educational level and C-latency located in the temporal region (T8, TP7) was presented as *P* = 0.035 r = −0.386; *P* = 0.023 r = −0.415 (Figure 7).

#### 3.6.4. Evaluation between clinical course and iCNV-latency

**Figure 8:**
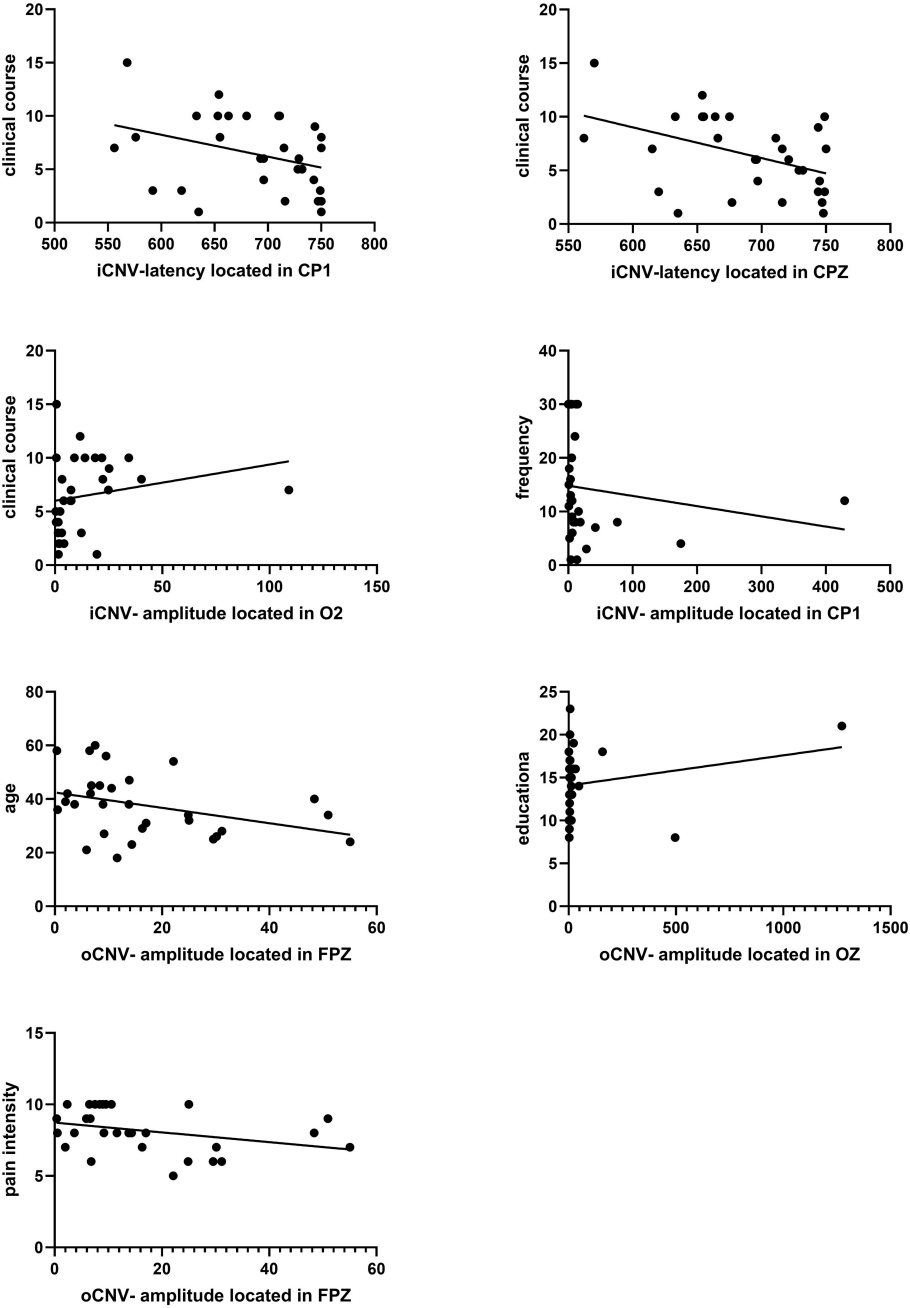
Correlation analysis between iCNV indicators and clinical course frequency. And correlation analysis between oCNV-amplitude and age, education, and pain intensity. A significant correlation between clinical course and iCNV-latency located in CP1 or CPZ was found (*P* = 0.043 r = −0.372, *P* = 0.031 r = −0.394). A significant correlation between clinical course and iCNV-amplitude located in O2 was found (*P* = 0.048 r = 0.364). A significant correlation between frequency and iCNV-amplitude located in CP1 was found (*P* = 0.026 r = −0.406). A significant correlation between age and oCNV-amplitude located in FPZ was found (*P* = 0.016 r = −0.436). A significant correlation between education and oCNV-amplitude located in OZ was found (*P* = 0.039 r = −0.378). A significant correlation between pain intensity and oCNV-amplitude located in the FPZ was found (*P* = 0.027 r = −0.402).

There was a significant negative correlation between clinical course and iCNV-latency, which was distributed in the central region (CP1, CPZ). The Pearson coefficient was presented as *P* = 0.043 r = −0.372; *P* = 0.031 r = −0.394 (Figure 8). Table 2 summarizes Pearson's correlation results between pain severity, clinical course, duration, education, age, and CNV-latency.

**Table 2:** Pearson's correlation results between pain severity, clinical course, duration, education, age and CNV-latency.

**ERP**	**Pain severity**				**Clinical course**				**Duration**				**Education**				**Age**			
	**L**	***P***	***r***	**95% CI**	**L**	***P***	***r***	**95% CI**	**L**	***P***	***r***	**95% CI**	**L**	***P***	***r***	**95% CI**	**L**	***P***	***r***	**95% CI**
A-latency	F3	0.02^*^	−0.423	−0.666/−0.049	T8	0.04^*^	−0.377	−0.679/−0.072	T8	0.036^*^	−0.384	−0.669/−0.055	-	-	-	-	T8	0.02^*^	0.423	0.122/0.705
	CP1	0.014^*^	−0.443	−0.659/−0.036	CPZ	0.031^*^	−0.393	−0.678/−0.071	-	-	-	-	-	-	-	-	-	-	-	-
	CPZ	0.038^*^	−0.381	−0.668/−0.052	CP2	0.01^**^	−0.464	−0.678/−0.071	-	-	-	-	-	-	-	-	-	-	-	-
	PZ	0.038^*^	−0.381	−0.673/−0.062	P4	0.042^*^	−0.373	−0.671/−0.058	-	-	-	-	-	-	-	-	-	-	-	-
	P4	0.006^**^	−0.489	−0.602/0.058	-	-	-	-	-	-	-	-	-	-	-	-	-	-	-	-
	O2	0.008^**^	−0.476	−0.617/0.035	-	-	-	-	-	-	-	-	-	-	-	-	-	-	-	-
C-latency	-	-	-	-	-	-	-	-	FP2	0.029^*^	0.399	−0.050/0.607	T8	0.025^*^	−0.407	−0.669/−0.055	-	-	-	-
	-	-	-	-	-	-	-	-	F3	0.04^*^	0.376	0.005/0.641	TP7	0.023^*^	−0.415	−0.725/−0.161	-	-	-	-
	-	-	-	-	-	-	-	-	C4	0.01^**^	0.465	0.017/0.648	-	-	-	-	-	-	-	-
	-	-	-	-	-	-	-	-	PZ	0.025^*^	0.408	−0.017/0.628	-	-	-	-	-	-	-	-
	-	-	-	-	-	-	-	-	O1	0.016^*^	0.437	0.059/0.671	-	-	-	-	-	-	-	-
iCNV-latency	-	-	-	-	CP1	0.043^*^	−0.372	−0.629/0.014	-	-	-	-	-	-	-	-	-	-	-	-
	-	-	-	-	CPZ	0.031^*^	−0.394	−0.689/−0.091	-	-	-	-	-	-	-	-	-	-	-	-

* *P* < 0.05,

** *P* < 0.01, L, location; in the 95% CI column, before the “/” is the lower limit and after the “/” is the upper limit.

#### 3.6.5. Evaluation between clinical characteristics and iCNV-amplitude

There was a significant correlation between frequency or clinical course and iCNV-amplitude. The frequency of migraine attacks was correlated with iCNV-amplitude located in CP1. The Pearson coefficient was presented as *P* = 0.026 r = −0.406. The clinical course was related to iCNV-amplitude located in O2, and the Pearson coefficient was presented as *P* = 0.048 r = 0.364 (Figure 8).

#### 3.6.6. Evaluation between clinical characteristics and oCNV-amplitude

The oCNV amplitude was correlated with age, educational level, and pain intensity. Individually, the Pearson coefficient between age and oCNV-amplitude located in FPZ was presented as *P* = 0.016 r = −0.436; the Pearson coefficient between educational level and oCNV-amplitude located in OZ was presented as *P* = 0.039 r = −0.378; the Pearson coefficient between pain intensity and oCNV-amplitude located in FPZ was presented as *P* = 0.027 r = −0.402 (Figure 8). Table 3 summarizes Pearson's correlation results between pain severity, clinical course, frequency, education, age, and CNV-amplitude.

**Table 3:** Pearson's correlation results between pain severity, clinical course, frequency, education, age and CNV-amplitude.

**ERP**	**Pain severity**				**Clinical course**				**Frequency**				**Education**				**Age**			
	**L**	***P***	***r***	**95% CI**	**L**	***P***	***r***	**95% CI**	**L**	***P***	***r***	**95% CI**	**L**	***P***	***r***	**95% CI**	**L**	***P***	***r***	**95% CI**
iCNV-amplitude	-	-	-	-	O2	0.048^*^	0.364	−0.173/0.522	CP1	0.026^*^	−0.406	−0.495/0.209	-	-	-	-	-	-	-	-
oCNV-amplitude	FPZ	0.027^*^	−0.402	−0.623/0.025	-	-	-	-	-	-	-	-	OZ	0.039^*^	0.378	−0.152/0.538	FPZ	0.016^*^	−0.436	−0.639/−0.002
tCNV-amplitude	FPZ	0.047^*^	−0.365	−0.411/0.307	-	-	-	-	-	-	-	-	-	-	-	-	-	-	-	-

* *P* < 0.05,

L, location; in the 95% CI column, before the “/” is the lower limit and after the “/” is the upper limit.

### 3.7. Multiple regression analyses in the migraine group

#### 3.7.1. Multiple linear regression analysis between the migraine-related indicators and A-latency

**Figure 9:**
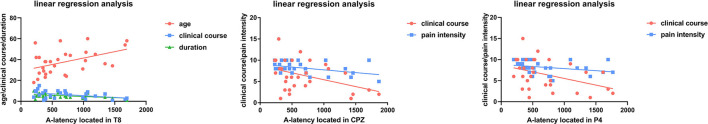
Linear regression analysis between clinical course, duration, age, pain severity, and A-latency.

We performed multiple linear regression analyses between the migraine-related indicators and A-latency. The results showed that there was a significant correlation between age and the A-latency located in the temporal area (T8) (Figure 9). Additionally, the A-latency was affected by age individually as a positive factor (β = 0.368, *P* = 0.033), while the A-latency was not affected by clinical course or attack duration (β=-0.177, *P* = 0.323; β = −0.307, *P* = 0.080), as shown in Table 4.

**Table 4:** The regression analysis between clinical features and the A-latency located in T8, CPZ, P4.

**Position**	**Clinical features**	**β**	**t**	***p***	**95% confidence interval**	
					**Lower limits**	**Upper limits**
T8	Clinical course	−0.307	−1.822	0.080	−80.511	4.857
	Duration	−0.177	−1.008	0.323	−97.703	33.388
	Age	0.368	2.253	0.033	1.209	26.390
CPZ	Clinical course	−0.350	−2.095	0.046	−90.144	−0.935
	Pain severity	−0.330	−1.975	0.059	−208.906	3.996
P4	Clinical course	−0.360	−2.070	0.048	−93.236	−0.409
	Pain severity	−0.231	−1.329	0.195	−182.527	39.006

^*^P < 0.05, ^**^P < 0.01.

The results showed that the A-latency located in CPZ was affected by clinical course individually (β = −0.350, *P* = 0.046), while the pain intensity was not affected (β = −0.330, *P* = 0.059) (Figure 9), as shown in Table 4.

The results showed that the A-latency located in P4 was affected by the clinical course individually (β = −0.360, *P* = 0.048), while the pain intensity was not affected (β = −0.231, *P* = 0.195) (Figure 9), as shown in Table 4.

## 4. FFT data

Fast Fourier Transformation (FFT) was applied to complete spectral analysis on all channels to analyze four-spectrum power. The beta was defined as a fast EEG activity of 14–30 Hz; the alpha was defined as 7.5–12.5 Hz EEG activity; the theta was defined as 4–7.5 Hz EEG activity, and the delta was defined as 1.5–4 Hz EEG activity.

According to our results, the delta or theta activity in the migraine group was statistically decreased compared to the control group, but there was no difference in beta or alpha activity between the migraine group and the control group.

### 4.1. Delta activity distributions

#### 4.1.1. The distributions of delta activity in different cortex regions

**Figure 10:**
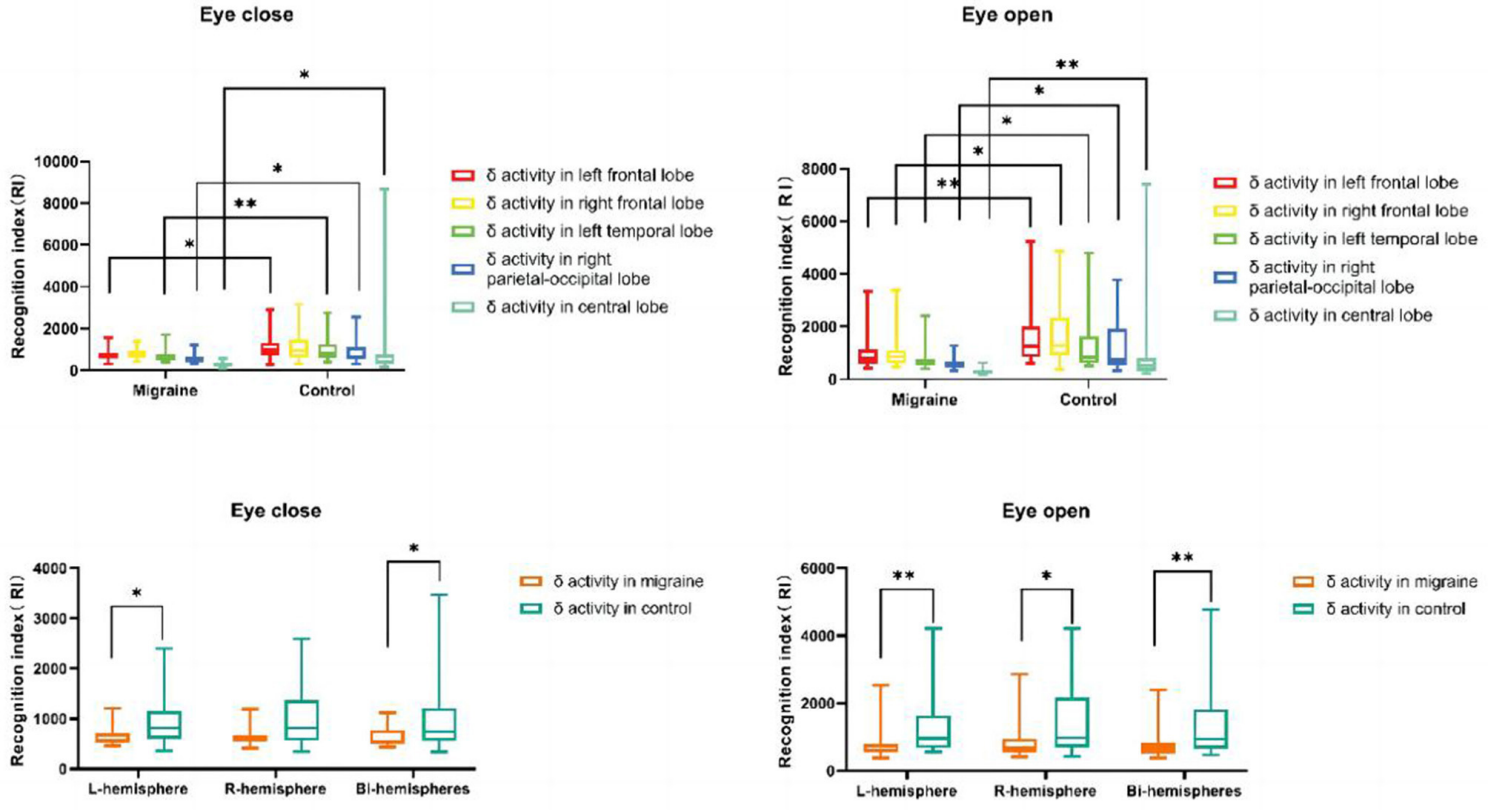
δ activity distributions in different cortex regions. δ activity distributions in the unilateral or bilateral hemisphere (*P < 0.05, **P < 0.01).

Regardless of eye-closed or eye-open conditions, delta activity in the migraine group located in the left frontal lobe was decreased compared to the control group, especially in the eye-open condition (*P* = 0.004). In the eye-open condition, there was a significant difference in delta activity located in the right frontal lobe between both groups (*P* = 0.013). Regardless of eye-closed or eye-open conditions, delta activity was significantly decreased in the left temporal lobe in the migraine group compared to the control group, especially in eye-close conditions (*P* = 0.007). Regardless of eye-close or eye-open conditions, delta activity located in the right parietal-occipital lobe (*P* = 0.019) or central lobe (*P* < 0.001) was decreased significantly compared to the control group, especially in eye-open conditions (Figure 10).

#### 4.1.2. The distribution of delta activity in the unilateral or bilateral hemisphere

Delta activity located in bilateral hemispheres in the migraine group was significantly decreased compared to the control group, especially in eye-open conditions (*P* = 0.009). Additionally, Regardless of eye-closed or eye-open conditions, there was a significant difference in delta activity in the left hemisphere between the migraine and the control groups (*P* = 0.007). In the eye-open condition, delta activity in the right hemisphere decreased more than in the control group (*P* = 0.017) (Figure 10).

### 4.2. The distribution of theta activity

**Figure 11:**
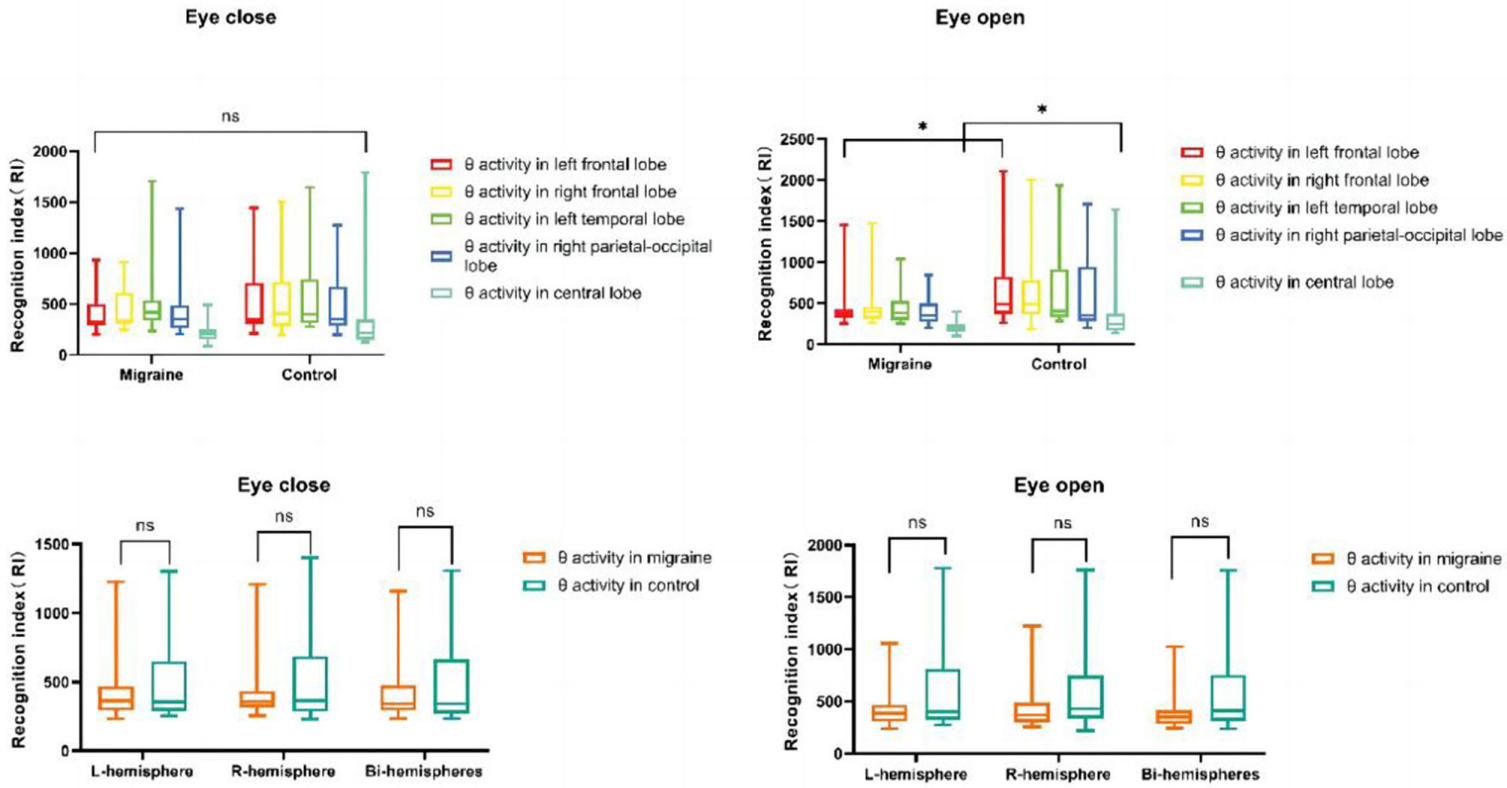
θ activity distributions in different cortex regions. θ activity distributions in the unilateral or bilateral hemisphere (*P < 0.05, **P < 0.01).

Only in eye-open conditions was a significant difference in theta activity observed in the left frontal lobe or central lobe between the migraine and control groups. Theta activity located in the frontal lobe was decreased (*P* = 0.038) and was similar in the central lobe (*P* = 0.038) (Figure 11). However, there was no difference in theta activity located in the bilateral or unilateral hemisphere between the migraine group and the control group (Figure 11).

## 5. Discussion

Migraine, a common primary neuropsychological disorder, often has cognitive impairments, such as attention, memory, and executive processing. CNV, as a slow cortical event-related potential, was associated with different stages of cognitive activity as interpreted by different electrophysiological components.

Our results were partly consistent with previous studies with longer CNV latency, higher CNV amplitude, or CNV-square in migraine participants. However, the behavioral difference was not statistically significant. Our study showed that migraine participants have the same hit rate as healthy subjects but a little longer reaction time, which may indicate poor responsiveness and execution in migraine patients.

As the results showed, migraine patients had significantly longer A-latency, C-latency, and iCNV-latency than healthy subjects. The statistical difference was mostly distributed throughout the whole brain. A-latency, located in the occipital area, and iCNV-latency, located in the prefrontal area, were the most significant individually, while C-latency was distributed all over the brain. Additionally, higher CNV amplitude was only distributed in the parietal or occipital brains. As interpreted by electrophysiological data, the longer CNV latency may indicate delayed response-ability. For example, Siniatchkin M believed that the early component iCNV was a critical electrophysiological indicator associated with stimulus processing, such as attention and preparation (17).

As the results of our study show, the longer the early iCNV-latency, the slower the speed of attention or preparation to stimulus than in controls. Furthermore, the abnormal cortical responsivity observed in migraine by clinical neurophysiology fluctuates with the migraine cycle and attack frequency (21).

As for amplitudes, the results we obtained were similar to previous results (19). The maximum amplitude of iCNV, oCNV, or tCNV was presented during the interictal period and was significantly more negative (higher) than controls, which mainly focused on the parietal or occipital region, especially on the P4, O1, or O2 lobe. As for squares, the oCNV-square, the iCNV-square, the tCNV-square, or the PINV-square were larger than the controls, which revealed delayed habituation or reduced cortical inhibition in migraine. In addition, the differences in iCNV-square, tCNV-square, and oCNV-square were mainly loci in the occipital area. In a previous study, it was suggested that the higher CNV amplitude in migraine is related to the level of cortical excitability, especially in striatum-thalamocortical networks such as the thalamus, caudate premotor cortex, anterior cingulum, and frontal lobe cortex (22).

Additionally, tCNV-amplitude is related to estimation, stimulus anticipation, and motor processing, and oCNV-amplitude is related to habituation to repetitive stimuli (16, 17). When there is a lack of habituation, the level of pre-activation in the sensory cortex is reduced, and the oCNV-amplitude is elevated after repeated stimulation. Thus, with our results, it can be explained that higher amplitude revealed impaired processes of estimation and anticipation due to reduced levels of sensory cortex pre-activation or cortical hyper-excitability.

Indeed, a lack of habituation to visual and auditory stimuli has been widely accepted in migraine patients (6), which has been proven by our electrophysiological data. Habituation was defined as a fundamental function that allows the response system to weaken gradually with the repetition of the same stimulus. Thus, this function can help the brain remove meaningless or irrelevant information to ensure sustained attention, anticipation, and motor activity (23). A lack of habituation in migraine patients tends to indicate abnormal cortical excitability, which can be observed by increased amplitude, such as CNV or other event-related potential (24, 25).

In 1996, Schoenen et al. proposed the “ceiling” theory to clarify the lack of habituation in migraine patients due to reduced levels of pre-activation in the sensory cortex (11). According to this theory, healthy individuals with normal pre-activation in the sensory cortex readily reach the top of response activity after repeated sensory stimuli and show habituation. Migraineurs, starting from lower activation levels, have a large range of suprathreshold activation to the upper limit, which explains the lack of habituation after repetition of the same stimulus (12). Cortical hyper-excitability may result from overactivation of specific brain areas. According to imaging studies, auditory and visual cortices have been abnormally activated during migraine attacks due to inevitably increased cerebral blood flow (25).

In contrast, some researchers held that decreased pre-activation levels in migraine patients played another important role as well (22). The healthy subjects have normal pre-activation levels to ensure reasonable allocation of the cognitive resources to show habituation following a normal response. However, for migraine patients, their lower pre-activation level may indicate that the brain needs to allocate more cognitive resources to achieve a corresponding response to stimulus from the environment, and this redundant power is transformed into higher amplitudes in CNV after the repetition of the same stimulus. Such a process is unclear and complicated and might be mediated partly by the hypofunctional subcortical-cortical aminergic pathways (9), which need further research to explore. Thus, CNV, as an objective and sensitive electrophysiological tool, might be applied to detect such pre-activation and habituation functions in migraine patients.

In addition, FFT, interpreted as a pattern of power fluctuations, may be applied to learn the activation of the cortical nervous system (26, 27). FFT analysis would break the complicated original signal into sine waves of different frequencies. In our study, FFT analysis has been applied to explore the distribution of the abnormal spectrum across the cortex between migraine patients and healthy controls under different conditions, such as eye-open or eye-close. The results of the FFT analysis showed that the slow EEG, such as delta or theta activity, in migraine patients was significantly decreased compared to controls regardless of any stage, with three main distribution areas: the frontal, central, or right parietal-occipital area. This may be due to higher cortical excitability and redundant habituation deficiencies, which originated from diffusely increased cerebral blood flow (rCBF) during the interictal period in migraine patients (28).

Furthermore, multiple linear regression and correlation analyses were conducted between the clinical characteristics and CNV data, including age, clinical course, frequency, pain intensity, and duration, with multiple indicators. In the temporal location, age has been found to be an individual factor affecting the main trend of A-latency. It is undoubtedly true that healthy controls over 65 years have cognitive decline to varying degrees with increasing age, while the cognitive decline in migraine patients was found to be earlier due to the attack onset and age range (29). However, temporal location, clinical course, or migraine duration have no effect on A-latency. In central or parietal locations, the clinical course of episodic attacks was related to A-latency (30). Additionally, a moderate correlation between the clinical course and oCNV or iCNV amplitude located in the central and parietal areas has been found in our study, which is similar to previous studies with distribution in much more extensive areas (24).

Above all, there are still some limitations to our study. First, in our study, cortical inhibition or excitability has only been explored in migraine patients without aura, while we focus on the attack frequency but neglect the distinction between chronic and episodic attacks, which may indicate different habituation of the CNV. Thus, in further study, we should include different subtypes to present with separate discussions. Second, we should enlarge the sample size to verify the results obtained in this study. Third, the cortical dysfunction between the attack and the interval may be explored. Finally, CNV combined with imaging may be a better method to explore the pathophysiological mechanisms of migraine, with a promising future.

## 6. Conclusion

To sum up, this study has revealed that migraine attacks may influence responsivity, pre-activation, habituation, and cortical inhibition not only on the behavioral level but also on the electrophysiological level. Abnormal changes in cortical habituation and inhibition can be interpreted as CNV components. Additionally, correlation analysis has been found between age, clinical course, frequency, pain intensity, duration, and CNV components. Thus, repetitive migraine attacks attenuate cortical inhibition and impair executive function.

## Data availability statement

The raw data supporting the conclusions of this article will be made available by the authors, without undue reservation.

## Ethics statement

Ethical approval was not required for the studies involving humans because this type of research will not require ethical review in this unit for the duration of the project. The studies were conducted in accordance with the local legislation and institutional requirements. The participants provided their written informed consent to participate in this study.

## Author contributions

JN and YZ were involved in acquiring or analyzing and interpreting the data. YL has made substantial contributions to the conception, design, and drafting of the manuscript or revising it critically for important intellectual content. All authors provided their approval for the final version to be published. Each author participated sufficiently in the study to take public responsibility for appropriate portions of the content and agreed to be accountable for all aspects of the study, ensuring that questions related to the accuracy or integrity of any part of the work were appropriately investigated and resolved.

## Appendix

ERP machine: (Neuroscan, Curry 7, America); MATLAB:(R2019a, MathWorks, US).

## References

[1] Tolner EA Chen S Eikermann-Haerter K. Current understanding of cortical structure and function in migraine. Cephalalgia. (2019) 39:1683–99. 10.1177/0333102419840643 30922081 PMC6859601

[2] HCCotIHS (IHS). The International Classification of Headache Disorders, 3rd edition (beta version). Cephalalgia. (2013) 33:629–808. 10.1177/0333102413485658 23771276

[3] Santangelo G Russo A Trojano L Falco F Marcuccio L Siciliano M. Cognitive dysfunctions and psychological symptoms in migraine without aura: a cross-sectional study. J Headache Pain. (2016) 17:1–8. 10.1186/s10194-016-0667-0 27568039 PMC5002274

[4] Burstein R Noseda R Borsook D. Migraine: Multiple Processes, Complex Pathophysiology. J Neurosci. (2015) 35:6619–29. 10.1523/JNEUROSCI.0373-15.2015 25926442 PMC4412887

[5] Picton TW Bentin S Berg P Donchin E Hillyard SA Jr RJ. Guidelines for using human event-related potentials to study cognition: recording standards and publication criteria. Psychophysiology. (2000) 37:127–52. 10.1111/1469-8986.3720127 10731765

[6] Legrain V Janne P Laloux P Ossemann M Dupuis M Reynaert C. Clinical and pathophysiological contribution of event-related potentials used to study migraine headache. Rev Neurol. (2001) 157:365–75. 11398007

[7] Kamiya R. Analysis of cell vibration for assessing axonemal motility in Chlamydomonas. Methods. (2000) 22:383–7. 10.1006/meth.2000.1090 11133244

[8] Srinivasan N. Cognitive neuroscience of creativity: EEG based approaches. Methods. (2007) 42:109–16. 10.1016/j.ymeth.2006.12.008 17434421

[9] Ambrosini A Schoenen J. The electrophysiology of migraine. Curr Opin Neurol. (2003) 16:327–31. 10.1097/01.wco.0000073945.19076.1f 12858069

[10] Schoenen J Ambrosini A Sándor PS de Noordhout AM. Evoked potentials and transcranial magnetic stimulation in migraine: published data and viewpoint on their pathophysiologic significance. Clin Neurophysiol. (2003) 114:955–72. 10.1016/S1388-2457(03)00024-5 12804664

[11] Schoenen J. Deficient habituation of evoked cortical potentials in migraine: a link between brain biology, behavior and trigeminovascular activation? Biomed Pharmacother. (1996) 50:71–8. 10.1016/0753-3322(96)84716-0 8761712

[12] Brighina F Palermo A Fierro B. Cortical inhibition and habituation to evoked potentials: relevance for pathophysiology of migraine. J Headache Pain. (2009) 10:77–84. 10.1007/s10194-008-0095-x 19209386 PMC3451650

[13] Meyer B Keller A Wöhlbier H-G Overath CH Müller B Kropp P. Progressive muscle relaxation reduces migraine frequency and normalizes amplitudes of contingent negative variation (CNV). J Headache Pain. (2016) 17:1–9. 10.1186/s10194-016-0630-0 27090417 PMC4835398

[14] Böcker KB Timsit-Berthier M Schoenen J Brunia CH. Contingent negative variation in migraine. Headache. (1990) 30:604–9. 10.1111/j.1526-4610.1990.hed3009604.x 2262316

[15] Siniatchkin M Averkina N Andrasik F Stephani U Gerber W-D. Neurophysiological reactivity before a migraine attack. Neurosci Lett. (2006) 400:121–4. 10.1016/j.neulet.2006.02.019 16540242

[16] Bender S Resch F Weisbrod M Oelkers-Ax R. Specific task anticipation versus unspecific orienting reaction during early contingent negative variation. Clin Neurophysiol. (2004) 115:1836–45. 10.1016/j.clinph.2004.03.023 15261862

[17] Siniatchkin M Jonas A Baki H van Baalen A Gerber W-D Stephani U. Developmental changes of the contingent negative variation in migraine and healthy children. J Headache Pain. (2010) 11:105–13. 10.1007/s10194-009-0180-9 20013021 PMC3452294

[18] Kropp P Gerber WD. Contingent negative variation–findings and perspectives in migraine. Cephalalgia. (1993) 13:33–6. 10.1046/j.1468-2982.1993.1301033.x 8448786

[19] Kropp P Gerber WD. Contingent negative variation during migraine attack and interval: evidence for normalization of slow cortical potentials during the attack. Cephalalgia. (1995) 15:123–8. 10.1046/j.1468-2982.1995.015002123.x 7641246

[20] Kropp P Gerber WD. Prediction of migraine attacks using a slow cortical potential, the contingent negative variation. Neurosci Lett. (1998) 257:73–6. 10.1016/S0304-3940(98)00811-8 9865930

[21] Coppola G Schoenen J. Cortical excitability in chronic migraine. Curr. Pain Headache Rep. (2012) 16:93–100. 10.1007/s11916-011-0231-1 22076672

[22] Tian Q Xu S Guo Y Li J Han M Ma Y. Contingent negative variation for the periodicity of migraine attacks without aura. J Integr Neurosci. (2019) 18:269–76. 10.31083/j.jin.2019.03.193 31601075

[23] Groves PM Thompson RF. Habituation: a dual-process theory. Psychol Rev. (1970) 77:419–50. 10.1037/h0029810 4319167

[24] Di Clemente L Coppola G Magis D Fumal A De Pasqua V Di Piero V. Interictal habituation deficit of the nociceptive blink reflex: an endophenotypic marker for presymptomatic migraine? Brain. (2007) 130:765–70. 10.1093/brain/awl351 17251239

[25] Weiller C May A Limmroth V Jüptner M Kaube H Schayck RV. Brain stem activation in spontaneous human migraine attacks. Nat Med. (1995) 1:658–60. 10.1038/nm0795-658 7585147

[26] Zhang D Huang X Mao C Chen Y Miao Z Liu C. Assessment of normalized cerebral blood flow and its connectivity with migraines without aura during interictal periods by arterial spin labeling. J Headache Pain. (2021) 22:1–10. 10.1186/s10194-021-01282-y 34261444 PMC8278584

[27] Bai X Wang W Zhang X Hu Z Zhang Y Li Z. Cerebral perfusion variance in new daily persistent headache and chronic migraine: an arterial spin-labeled MR imaging study. J Headache Pain. (2022) 23:1–3. 10.1186/s10194-022-01532-7 36482334 PMC9733035

[28] Facco E Munari M Baratto F Behr AU Palù AD Cesaro S. Regional cerebral blood flow (rCBF) in migraine during the interictal period: different rCBF patterns in patients with and without aura. Cephalalgia. (1996) 16:161–8. 10.1046/j.1468-2982.1996.1603161.x 8734767

[29] Kohama SG Rosene DL Sherman LS. Age-related changes in human and non-human primate white matter: from myelination disturbances to cognitive decline. Age. (2012) 34:1093–110. 10.1007/s11357-011-9357-7 22203458 PMC3448998

[30] Kropp P Linstedt U Gerber W. Duration of migraine disease correlates with amplitude and habituation of event-related potentials. Schmerz. (2005) 19:489–92. 10.1007/s00482-005-0386-y 15756581

